# Transcriptome profiles reveal response mechanisms and key role of *PsNAC1* in *Pinus sylvestris* var. *mongolica* to drought stress

**DOI:** 10.1186/s12870-024-05051-2

**Published:** 2024-04-26

**Authors:** Chengcheng Zhou, Wenhao Bo, Yousry A. El-Kassaby, Wei Li

**Affiliations:** 1 State Key Laboratory of Tree Genetics and Breeding, National Engineering Research Center of Tree Breeding and Ecological Restoration, College of Biological Sciences and Technology, Beijing Forestry University, Beijing, 100083 China; 2 Department of Forest and Conservation Sciences, Faculty of Forestry, University of British Columbia, 2424 Main Mall, Vancouver, BC V6T 1Z4 Canada

**Keywords:** *Pinus sylvestris* var. *mongolica*, NAC transcription factor, Transcriptome, Drought stress

## Abstract

**Background:**

Drought stress severely impedes plant growth, and only a limited number of species exhibit long-term resistance to such conditions. *Pinus sylvestris* var. *mongolica*, a dominant tree species in arid and semi-arid regions of China, exhibits strong drought resistance and plays a crucial role in the local ecosystem. However, the molecular mechanisms underlying this resistance remain poorly understood.

**Results:**

Here, we conducted transcriptome sequence and physiological indicators analysis of needle samples during drought treatment and rehydration stages. *De-novo* assembly yielded approximately 114,152 unigenes with an N50 length of 1,363 bp. We identified 6,506 differentially expressed genes (DEGs), with the majority being concentrated in the heavy drought stage (4,529 DEGs). Functional annotation revealed enrichment of drought-related GO terms such as response to water (GO:0009415: enriched 108 genes) and response to water deprivation (GO:0009414: enriched 106 genes), as well as KEGG categories including MAPK signaling pathway (K04733: enriched 35 genes) and monoterpenoid biosynthesis (K21374: enriched 27 genes). Multiple transcription factor families and functional protein families were differentially expressed during drought treatment. Co-expression network analysis identified a potential drought regulatory network between cytochrome P450 genes (Unigene4122_c1_g1) and a core regulatory transcription factor Unigene9098_c3_g1 (*PsNAC1*) with highly significant expression differences. We validated *PsNAC1* overexpression in *Arabidopsis* and demonstrated enhanced drought resistance.

**Conclusions:**

These findings provide insight into the molecular basis of drought resistance in *P. sylvestris* var. *mongolica* and lay the foundation for further exploration of its regulatory network.

**Supplementary Information:**

The online version contains supplementary material available at 10.1186/s12870-024-05051-2.

## Background

Worldwide forests are subject to harsh and stressful environmental conditions [1] as their ecosystems expose to a range of weather extremes, including freezing temperatures during winter, high air temperatures during summer, and seasonal low water availability [2, 3]. More specifically, climate change has exacerbated water stress in many regions due to increased evaporative demand, altered precipitation patterns, and earlier snowmelt [4, 5]. Moderate drought commonly results in reduced growth and increased mortality [6, 7], and has a profound impact on the global distribution of plant communities [8–10]. As such, understanding the response of conifers to drought stress is a critical issue in global forest ecology.

Plants employ a range of biochemical, physiological, and molecular responses at the whole-plant, organ, tissue and cellular levels to cope with drought stress [11]. For instance, water deficiency can damage the basic structure of metabolites, inhibiting carbon assimilation and impairing photosynthetic activities, triggering a suite of various biochemical and physiological responses [12, 13]. In response to water scarcity, plants regulate stomatal conductance to maintain a consistent marginal water usage efficiency and prevent carbon gain [2]. While some general principles apply to both angiosperms and gymnosperms, there are notable differences between the two groups [5]. Gymnosperms tend to exhibit greater drought resistance due to lower stomatal sensitivity to vapor pressure deficit (VPD) and more cavitation-resistant xylem [14]. Conifer xylem consists solely of tracheids, whereas angiosperms may produce both tracheids and wide vessels, which have higher hydraulic conductivity but a smaller safety margin with respect to xylem pressures [14]. Further research is needed to determine whether the molecular mechanisms underlying the similar physiological responses of conifers and angiosperms to drought stress are the same, as well as to elucidate the molecular mechanisms underlying the distinct physiological responses observed in conifers.

Drought tolerance is a complex quantitative trait controlled by numerous genes involved in stress signal perception, signal transduction and amplification, and plant stress adjustments [3, 15, 16]. In response to drought stress, plants employ regulatory pathways that can be broadly categorized as ABA-dependent and ABA-independent [17]. Several transcription factors (TFs), including ABA-responsive element/ABRE binding factors (ABRE/ABF), MYBs, WRKYs, basic leucine zipper (bZIP) proteins, and NAC (NAM-ATAF-CUC2) proteins, play crucial roles in forming transcriptional networks that activate multiple biochemical and developmental pathways to enhance drought tolerance [16, 18]. NAC proteins belong to a large family of plant-specific transcription factors, many of which function in stress-response and developmental processes [19, 20]. Numerous stress-responsive NAC TFs have been overexpressed in plants to improve drought tolerance. For instance, overexpression of *TaNAC071-A* in wheat significantly enhanced drought tolerance through improved water-use efficiency and increased expression of stress-responsive genes [21]. Under drought stress, the survival rate of *GmNAC12*-overexpressed soybean lines increased by more than 57% compared to wild-type plants [22]. *Arabidopsis* lines overexpressing potato *StNAC053* displayed significant increased tolerance to salt and drought stress treatments [23]. Similar results have been reported in woody plants. Poplar plants overexpressing *PeNAC045* exhibited a drought-sensitive phenotype [24]. Poplar *PtrNAC006*, *PtrNAC007*, and *PtrNAC120*, regulated by *PtrAREB1*, were identified as positive regulators of drought response [25]. While the role of NAC TFs in regulating drought stress in angiosperms has been extensively studied, research on gymnosperms, particularly conifers, is still in its infancy. *PpNAC2* and *PpNAC3* encode stress-responsive NAC transcription factors involved in the jasmonate response in maritime pine [26]. *Picea wilsonii PwNAC11* activates ERD1 by interacting with ABF3 and DREB2A to enhance drought tolerance in transgenic *Arabidopsis* [27]. Overexpression of *PaNAC03*, a stress-induced NAC gene family transcription factor in Norway spruce, resulted in reduced flavonol biosynthesis and aberrant embryo development [28]. Systematic studies on the phenotype, physiology, gene expression, and regulatory networks of conifer NAC transcription factors in response to drought stress are needed.

*Pinus sylvestris* var. *mongolica* is naturally distributed in the northern mountains of the Greater Khingan Mountains and the Hulun Buir Sandy Steppe in China [29]. The species exhibits excellent characteristics such as cold, drought, and barren resistance, as well as rapid growth [30]. It is the primary tree species used for shelter, soil, and water conservation as well as timber production [29, 30]. *P. sylvestris* var. *mongolica* represents an ideal model for studying the molecular mechanisms of drought resistance in conifers. In a recent study, Meng et al. [31] explored the effects of soil drought stress on growth characteristics, root system, and tissue anatomy under different drought conditions, however, the molecular mechanisms involved remain poorly understood. Here, we subjected *P. sylvestris* var. *mongolica* seedlings to drought stress and rehydration, measured physiological indices during these processes, analyzed gene expression patterns during drought resistance using transcriptome sequencing, performed functional enrichment analysis of differentially expressed genes (DEGs), and further screened key transcription factors and functional proteins using differential expression levels and functional annotations. Among these, one NAC transcription factor was identified as a coregulated gene. Using *Arabidopsis thaliana* heterologous transgenesis, we demonstrated that this gene could enhance drought tolerance in *Arabidopsis*. These findings provide a foundation for understanding the molecular mechanisms of drought resistance in conifers and offer potential avenues for breeding drought-resistant conifers.

## Results

### Drought impact on photosynthesis and physiological indexes in *P. sylvestris* var. *mongolica*

**Fig. 1:**
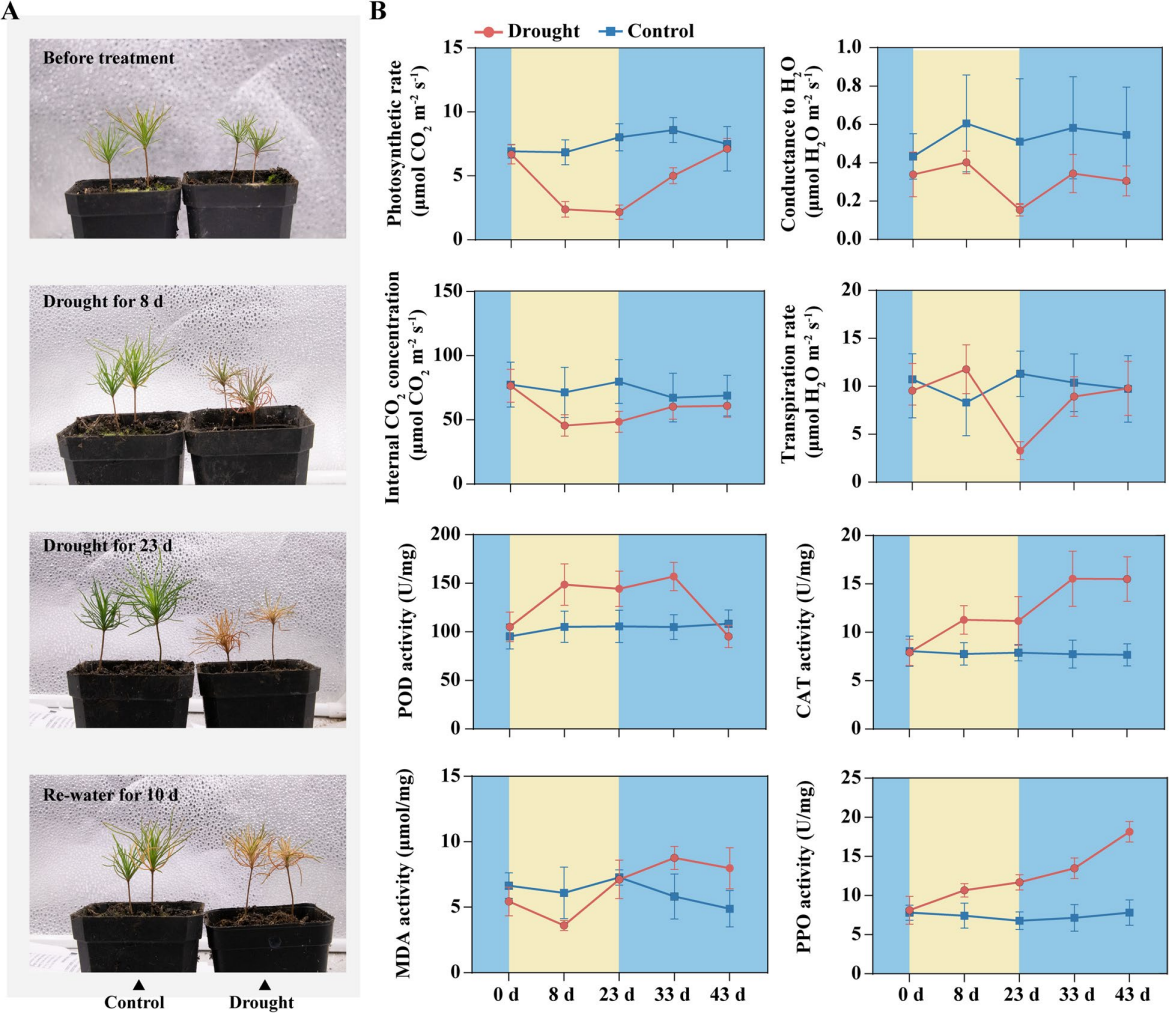
Phenotypic and physiological indices measurements of drought in *P. sylvestris* var. *mongolica*. **A** Phenotypes in mild drought (8 d), severe drought (23 d), and after rehydration (10 d). **B** Photosynthetic and enzymatic activity indicators

Under drought treatment, *P. sylvestris* var. mongolica seedlings exhibited mild drought at 8 days and severe drought at day 23, with some individuals failing to recover after rehydration (Fig. 1A). To investigate the effects of drought stress on photosynthesis and physiological parameters, including net photosynthesis (AN), stomatal conductance (Gs), internal CO2 concentration (Ci), and transpiration rate (E), these metrics were measured starting on the first day (day 0) prior to treatment. At mild drought (day 8), AN and Ci decreased significantly, but did not continue to decrease during the prolonged drought period until severe drought (day 23), remaining at approximately the same level as during mild drought (Fig. 1B). After rewatering, AN continued to increase and Ci stabilized after the 10th day of rewatering. In contrast, Gs and E were minimally affected during mild drought but decreased significantly during severe drought, both improving significantly after rewatering (Fig. 1B). The trends of these four indicators suggest that pine trees were recovering their growth status after rewatering. In the control group, AN and Gs tended to increase somewhat as the plants grew, while Ci and E remained stable (Fig. 1B).

Under stress, plants peroxidase (POD), malondialdehyde (MDA), catalase (CAT), and polyphenol oxidase (PPO) enzymes respond to stress. In our study, we measured these indicators and found that the total activities of POD, CAT, and PPO were significantly elevated compared to the control during the mild drought period (Fig. 1B). The total activities of CAT and PPO remained consistently higher than the control during both the drought and rehydration phases (Fig. 1B). POD activity recovered to the same level as the control after 20 days of rehydration. In contrast, MDA levels decreased during the mild drought period, but began to rise during severe drought and continued to increase after rehydration (Fig. 1B). Overall, all four enzyme activity indicators were generally higher than the control during both the drought treatment and rewatering stages, indicating that these enzymes play an important role in the drought stress and recovery stages of *P. sylvestris* var. *mongolica*.

### Global transcriptomic response to drought in *P. sylvestris* var. *mongolica*

**Fig. 2:**
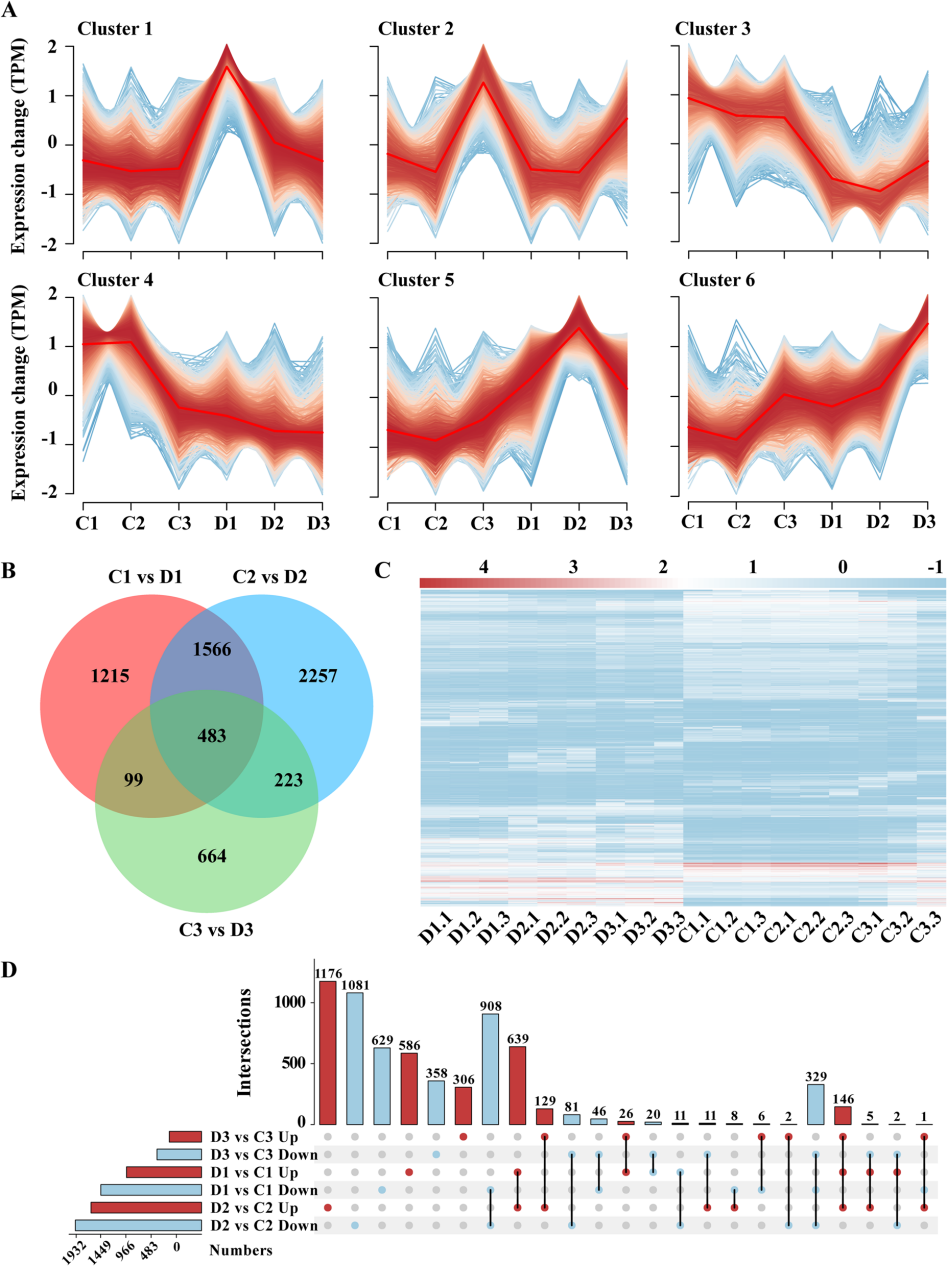
Global transcriptomic response to drought in *P. sylvestris* var. *mongolica*. **A** Trend classification map of gene expression. **B** VENN plots of DEGs in different treatment stages. **C** Overall expression heatmap of DEGs. **D** UPSET plots of DEGs in different treatment stages

To investigate gene expression in *P. sylvestris* var. *mongolica* under drought treatment, transcriptome sequencing of the drought-treated and well-watered samples was performed. After filtering out adapter sequences and reads ≤ 50 bp, 520,258,556 and 563,999,036 clean data were obtained from the control and drought treatments, respectively (Table 1). A total of 114,152 unigenes (mean length 910.42 bp, N50 length 1,363 bp; Table 2) were obtained by de novo assembly with average GC content of 41.07%. The expression trends of all unigenes could be classified into six categories (Fig. 2A). Overall, Cluster 1, Cluster 5, and Cluster 6 had an up-regulated unigenes trend during drought, while Cluster 3 and Cluster 4 had down-regulated unigenes trend and Cluster 2 had nonsignificant trend. Among them, unigenes in Cluster 1 were significantly up-regulated in expression during mild drought and decreased during severe drought and rehydration stages, suggesting that the genes in Cluster 1 may play an important role in short time response to drought stress. Unigenes in Cluster 5 were continuously up-regulated during mild and severe drought and decreased during rehydration stages, indicating that they continuously responded during drought stages. Unigenes in Cluster 6, on the other hand, were continuously up-regulated during the drought and rehydration phases, and they may have a role in both the drought resistance and growth recovery phases.

**Table 1:** Summary of BGISEQ sequencing of *P. sylvestris* var. *mongolica* under drought treatment

	**Control**	**Drought**
Number of total raw reads	534,207,632	577,017,556
Number of total clean reads	520,258,556	563,999,036
Average effective rate (%)	97.43	97.75
Average error rate (%)	0.02	0.02
Average Q20 (%)	98.17	98.23
Average Q30 (%)	94.14	94.33
Average GC content (%)	44.85	45.40

**Table 2:** Transcriptomic de novo assembly statistics

Contig category	Number (bp)
N10	4,206
N20	3,122
N30	2,416
N40	1,866
N50	1,363
Median length	525
Average length	910.42

From the differential expression analysis of two-by-two comparison of drought treatment and control, 6,506 differential genes (concatenated sets; Fig. 2C) were obtained, with 3363 differential genes in C1vsD1, 4,529 differential genes in C2vsD2, and 1469 differential genes in C3vsD3, indicating that *P. sylvestris* var. *mongolica* genes had a pronounced response during severe drought. Additionally, 483 genes were differentially expressed in all three stages (Fig. 2B), suggesting that these genes may be the core genes in the drought resistance process of pine, among which 329 exhibited a down-regulated expression trend (Fig. 2D). These genes may be involved in growth and metabolic processes that are suppressed during drought stress.

### Enrichment analysis is closely related to drought stress

**Fig. 3:**
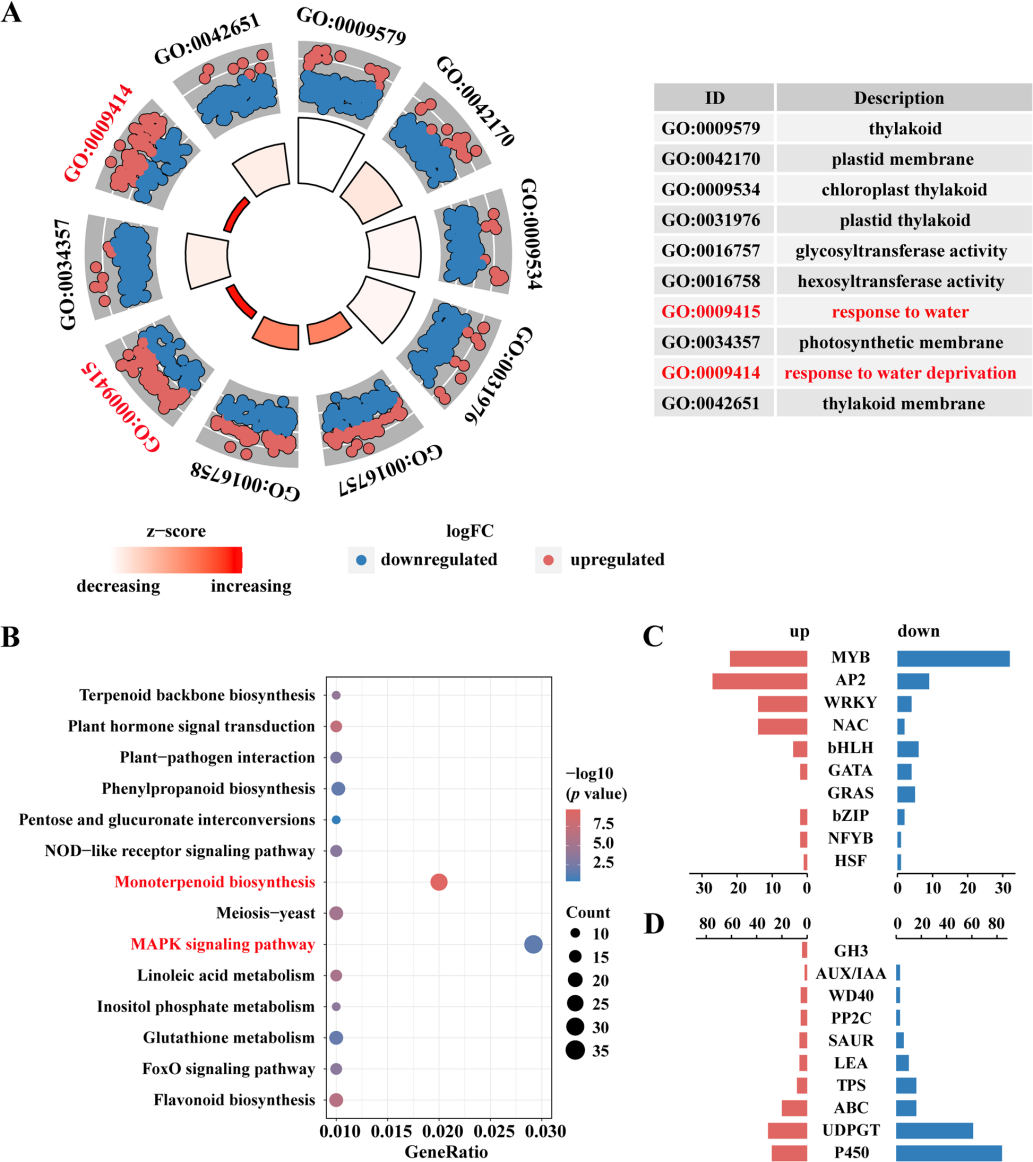
Enrichment analysis and functional classification of DEGs. **A** GO enrichment analysis of DEGs. **B** KEGG enrichment analysis of DEGs. **C** Classification and differential expression of transcription factors in DEGs. **D** Classification and differential expression of functional protein in DEGs

To determine the main biological functions and pathways of all significantly differentially expressed genes (DEGs), gene ontology and pathway annotation were performed. GO and KEGG enrichment analyses were conducted using the annotation information of differential genes. The results of GO showed that a total of 2,030 DEGs were annotated in 248 GO terms, including 171 biological processes (BP), 57 molecular functions (MF), and 19 cellular components (CC). The top 10 GO terms with the highest enrichment numbers were selected for further analysis (Fig. 3A), revealing that two terms were associated with drought stress: response to water (GO:0009415) with 108 genes and response to water deprivation (GO:0009414) with 106 genes, indicating that a large number of genes are regulated by drought stress. All DEGs were annotated in 113 KEGG categories, and the top 14 categories with the highest enrichment numbers were selected for further analysis (Fig. 3B). Among the KEGG terms with a high number of enriched genes were two pathways: MAPK signaling pathway (K04733: 35 genes), an important signaling pathway in plant abiotic stress response signaling; and monoterpenoid biosynthesis (K21374: 27 genes), which is also associated with response to stress.

### Differential expression of transcription factors and functional proteins involved in drought stress

All genes were functionally annotated, of which 29,286 genes were annotated to function, including 3,718 differential genes. It was found that 149 genes were classified as transcription factors (TFs). These TFs comprised 10 families, including MYB (54 genes), AP2 (36 genes), WRKY (18 genes), NAC (16 genes), bHLH (10 genes), GATA (6 genes), GRAS (5 genes), bZIP (4 genes), NF-YB (3 genes) and HSF (2 genes). The expression trends of different transcription factor families varied, with the largest number of MYB having more down-regulated genes (32 genes) than up-regulated genes (22 genes), while AP2, WRKY and NAC had significantly more up-regulated than down-regulated genes (Fig. 3C). Different transcription factor families may play different roles in drought stress. In addition to the regulatory role of transcription factors, the expression of downstream functional proteins during stress is critical in influencing plants physiological and biochemical responses. Among the differential genes, all 3569 were functional proteins except for 149 transcription factors. Ten functional protein families associated with drought stress were screened and most were down-regulated (Fig. 3D). Among them, 84 P450 family members were down-regulated, accounting for 75.0% of the 112 family members. Additionally, 61 UDPGT family members were down-regulated in expression, accounting for 66.3% of the 92 family members. It is likely that the down-regulation of these family members is related to growth inhibition and development after stress.

Notably, 24 Terpene synthesis (TPS) genes were screened, of which 16 were down-regulated and 8 were up-regulated (Fig. 3D). TPS genes are key rate-limiting enzymes for terpene synthesis, catalyzing the production of different terpenoids from terpenoid substrates. Combined with the significant enrichment of the monoterpene synthesis pathway in the results of KEGG analysis, this suggests that terpenoid synthesis is significantly altered in Pinus sylvestris after drought stress and that these terpenoids may be involved in drought resistance.

### Identification of hub genes associated with control, drought stress, and recovery *in P. sylvestris var. mongolica*

**Fig. 4:**
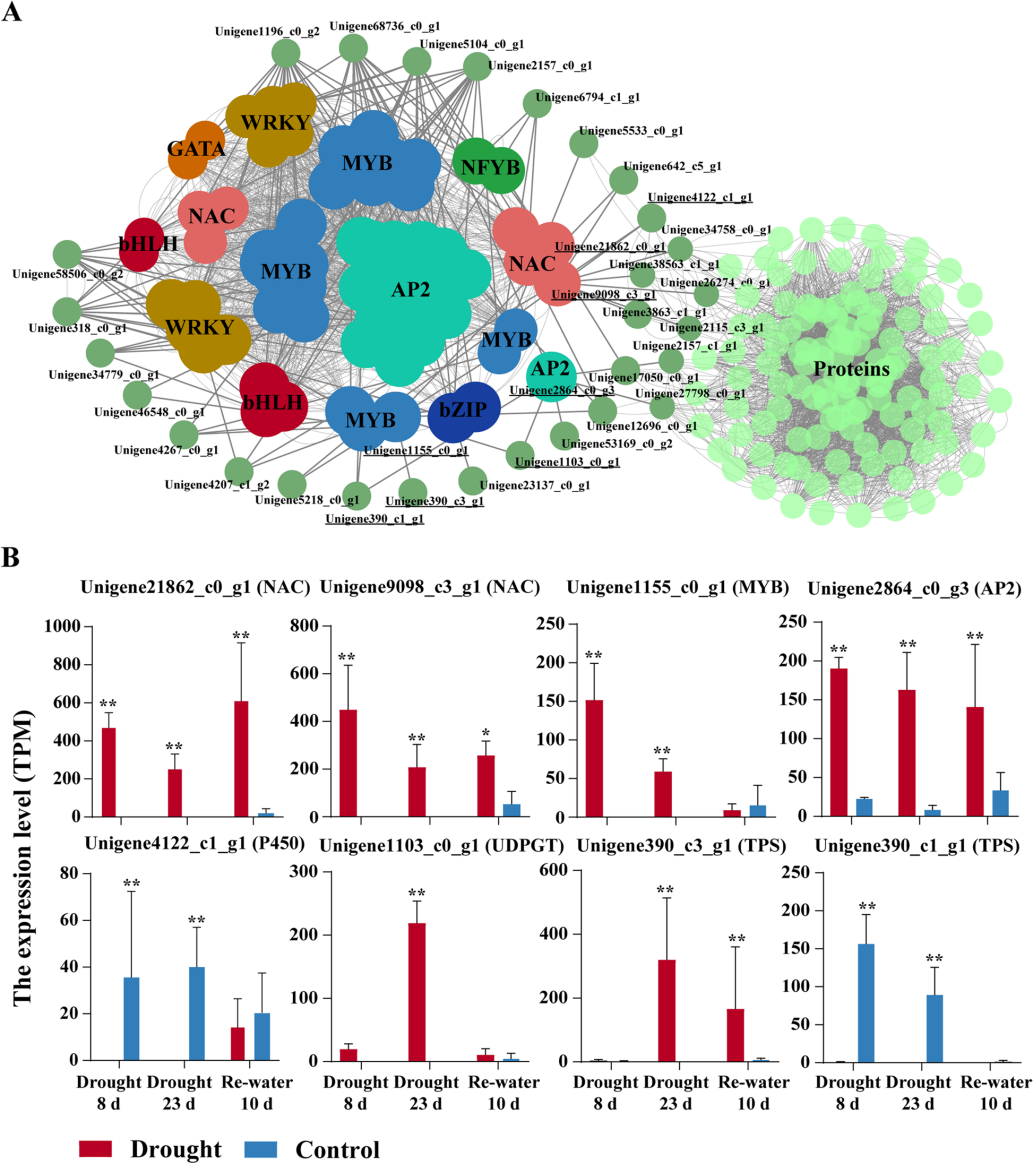
Co-expression network construction of DEGs and the expression level of key genes. **A** Co-expression network construction of DEGs. **B** Expression level of Unigene21862_c0_g1, Unigene9098_c3_g1, Unigene1155_c0_g1, Unigene2864_c0_g3, Unigene4122_c1_g1, Unigene1103_c0_g1, Unigene390_c3_g1, and Unigene390_c1_g1. Data from three independent biological replicates were shown with standard error (SE). * and ** indicate significant difference and highly significant difference, respectively (t-test was used for significance analysis)

To accurately screen for key differential genes, a high-sensitivity threshold (log2FoldChange ≥ 2, *P* ≤ 0.01) was used to identify 2,802 (43.1%) significantly DEGs. In order to be able to screen for positively regulated transcription factors that enhance drought resistance, these significantly DEGs were subjected to Pearson correlation analysis and screened using a threshold (R ≥ 0.8) to obtain 917 gene pairs. Co-expression networks were constructed using these gene pairs and visualized using Cytoscape (Fig. 4A). The co-expression network revealed more associations were between transcription factors and functional proteins, and relatively fewer associations between transcription factors and functional proteins. The Cytoscape CytoNCA tool was used to analyze hub transcription factors, which were then filtered using foldchange to identify four hub transcription factors: Unigene21862_c0_g1 (NAC), Unigene9098_c3_g1 (NAC), Unigene1155_c0_g1 (MYB), and Unigene2864_c0_g3 (AP2). The functional proteins with their four potential co-expression relationships were Unigene4122_c1_g1 (P450), Unigene1103_c0_g1 (UDPGT), Unigene390_c3_g1 (TPS), and Unigene390_c1_g1 (TPS). The expression of Unigene1103_c0_g1 (UDPGT), Unigene390_c3_g1 (TPS), and four TFs were significantly up-regulated in the drought treatment, while the expressions of P450 and TPS1 was significantly down-regulated (Fig. 4B). Among them, Unigene9098_c3_g1 (NAC, named as *PsNAC1*) had the highest FC of all genes and almost had no expression in WT, while the mean expression TPM was 449.77 in drought-treated and rehydrated stages (Fig. 4B), which was highly significant. NAC has been widely reported to be associated with stress response in angiosperms, so *PsNAC1* may play a central regulatory role in drought resistance in *P. sylvestris* var. *mongolica* and could be a candidate gene for subsequent validation.

### A key hub gene *PsNAC1* enhanced *A. thalinana* drought tolerance

**Fig. 5:**
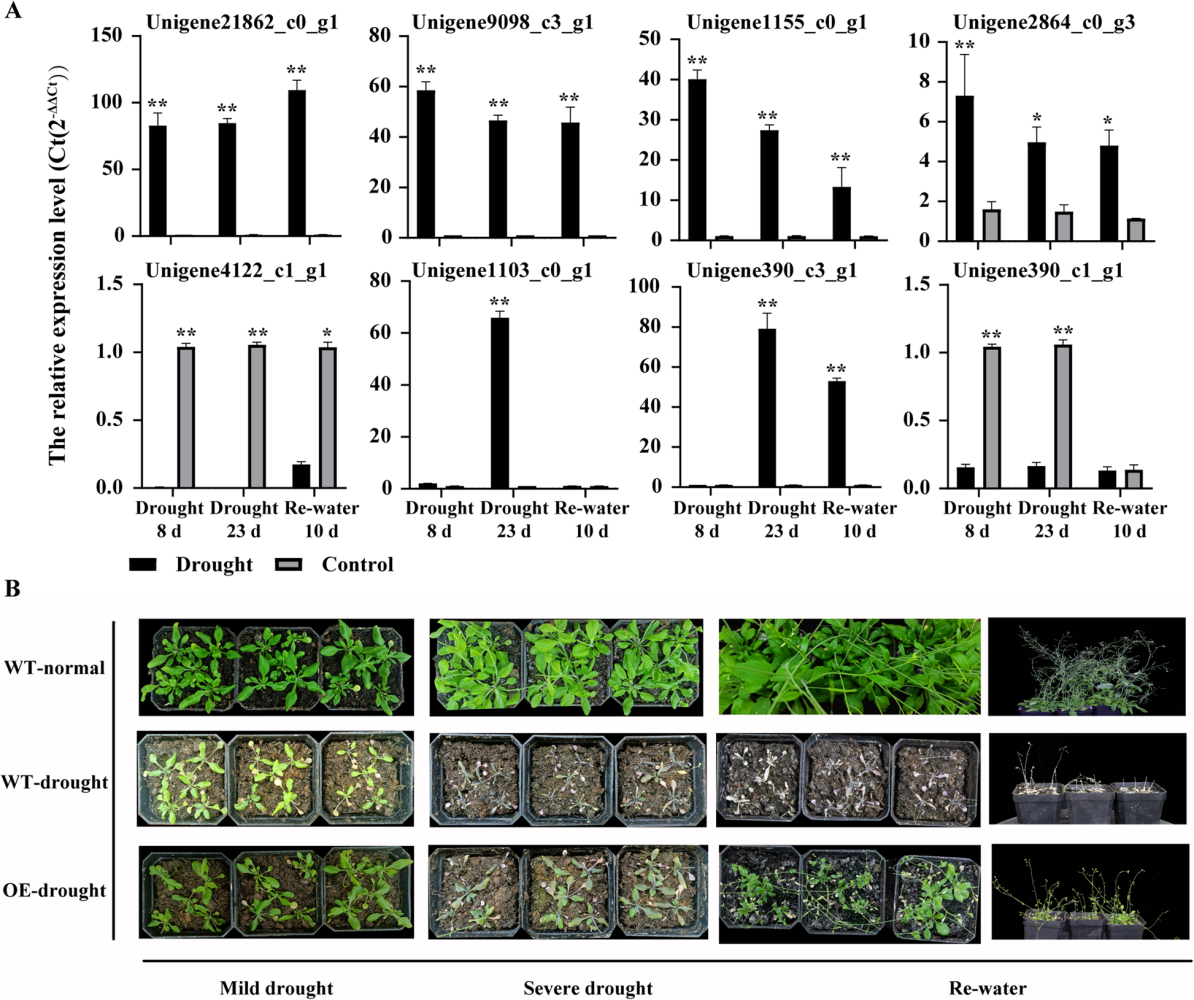
The qPCR validation of key gene expression and overexpression phenotype of *PsNAC1* in *Arabidopsis thaliana*. **A** The qPCR validation of Unigene21862_c0_g1, Unigene9098_c3_g1, Unigene1155_c0_g1, Unigene2864_c0_g3, Unigene4122_c1_g1, Unigene1103_c0_g1, Unigene390_c3_g1, and Unigene390_c1_g1. **B** Overexpression phenotype of *PsNAC1* in *Arabidopsis thaliana*. Data from three independent biological replicates were shown with standard error (SE). * and ** indicate significant difference and highly significant difference, respectively (t-test was used for significance analysis)

To validate the transcriptome data of the above eight genes, the expression of these genes in the drought treatment and control was verified by qPCR (Fig. 5A). The results showed that the relative expression trends of the eight genes were generally consistent with the transcriptome data. If the TPM of a given gene in the transcriptome was less than 5 at a certain stage, its relative expression was essentially undetectable by qPCR. Additionally, genes with expression at the rehydration stage were also not detected by qPCR, which may be due to experimental error arising from the fact that the transcriptome and qPCR samples were not derived from the same plants.

To verify the function of the key regulatory gene *PsNAC1*, an overexpression vector for this gene was constructed and stably transferred into *Arabidopsis* to obtain T2 generation positive seedlings after drought treatment. The results showed that the overexpressed positive seedlings exhibited stronger drought resistance compared to wild-type plants (Fig. 5B). Overexpressed plants displayed a later onset of mild drought phenotype and were able to partially recover their growth after rehydration following severe drought. These findings suggest that *PsNAC1* enhances drought resistance and is a key gene in the drought resistance process of *P. sylvestris* var. *mongolica*.

## Discussion

Plants produce a series of physiological and biochemical responses when subjected to drought stress, which can be broadly categorized into three phases: drought avoidance, resistance, and resilience [5, 14, 32, 33]. Previous studies and our results have shown that changes in major photosynthetic physiology, enzyme activity responses, and growth indicators in response to drought are similar between conifers and angiosperms under mild and severe drought [2, 5, 26, 28, 31, 34]. For instance, photosynthetic capacity decreases significantly with increasing drought, while the level of antioxidant enzyme activities increases significantly. Downregulating photosynthesis may be a form of drought adjustment to protect the hydraulic system [35], or to limit the metabolic costs of maintaining the photosynthetic machinery needed to generate high photosynthetic outputs when environmental conditions are unfavorable [36, 37]. The substantial upregulation of antioxidant enzyme activity serves to avoid membrane damage by reactive oxygen species [37, 38]. General indicators do not appear to be optimal for probing the response of conifers to drought stress. Subsequent studies could focus on tissue organs unique to gymnosperms, such as resin canals and secondary xylem, which consists almost entirely of tracheids. In *Pinus sylvestris*, drought-induced xylem embolism and reduced water-transport capacity limit the recovery of leaf gas exchange [13]. Further investigation is warranted to understand how conifers utilize unique tissue structures and physiological responses to achieve growth recovery despite rehydration after experiencing severe drought.

Under various stress conditions, changes in the expression of various genes can result in different physiological and biochemical processes, and studying molecular mechanisms is an important aspect of in-depth research on the mechanisms of stress tolerance. Transcription factor-based engineering has been used as a powerful tool for improving stress tolerance in angiosperms [39]. However, with the exception of a few gymnosperms such as *Pinus tabuliformis* [40], *Ginkgo biloba* [41], and *Sequoiadendron giaganteum* [42], which have chromosome-level genome sequences, the vast majority of gymnosperms lack genome sequences and complete gene annotations. As a result, de novo assembly and homology annotation using transcriptomes is currently a common approach for studying transcription factors in conifers. In this study, transcriptome sequencing was used to analyze gene expression patterns during mild drought, severe drought, and rehydration stages of Pinus sylvestris. A key NAC transcription factor (Unigene9098_c3_g1, *PsNAC1*) was identified through bioinformatics analysis such as differential expression, correlation, and co-expression network analysis combined with functional annotation of homologous genes. Reciprocal BLAST analysis revealed that the protein sequence identity of *PsNAC1* with *PwNAC11*, *ANAC032*, and *GmNAC2* was 84, 72, and 67%, respectively, indicating both sequence conservation and diversification. Gene sequences that are highly homologous to some extent may have similar gene functions. *ANAC032* overexpression lines exhibited enhanced leaf senescence when challenged with different oxidative (3-aminotriazole, fumonisin B1, and high light) and abiotic stress (osmotic and salinity) conditions compared to the wild type [43]. *GmNAC2* was also strongly induced by osmotic stress [44]. Interestingly, compared to the wild type, *PsNAC1* showed an eightfold up-regulation of gene expression under drought stress, with the highest differential multiplicity of all transcription factors. It was highly expressed at all three stages of treatment but was not expressed at any stage in the wild type. Heterologous transgenic *Arabidopsis* also showed improved drought tolerance. These results suggest that *PsNAC1* is a key regulatory gene in drought stress in *P. sylvestris* var. *mongolica*.

The NAC transcription factor family has been shown to be associated with drought tolerance in both angiosperms and conifers [20, 24, 26, 28, 42]. For example, overexpression of *PwNAC2* in *Arabidopsis* resulted in a more vigorous seed germination and significant tolerance through ROS scavenging, reduced membrane damage, slower water loss, and increased stomatal closure [28]. However, these results do not indicate that the pathways regulated by these NAC genes are the same in both angiosperms and conifers, and the existence of unique regulatory pathways in conifers remains unclear. It is worth noting that both differential expression and GO/KEGG enrichment analysis in our study focused on the terpenoid synthesis pathway. Meanwhile, the core co-expression network contains several terpene synthases (TPS) and cytochrome P450 (P450). TPS protein family members are capable of synthesizing monoterpenoids, sesquiterpenoids, diterpenoids, triterpenoids, tetraterpenoids, and their derivatives using products from the MVA and MEP pathways as substrates [35, 45], while cytochrome P450 catalyzes the conversion of terpenes to resin acids [46, 47]. In angiosperms, numerous studies in angiosperms have shown that TPS genes can be induced by biotic and abiotic stresses and promote terpenoid synthesis [48–50], including *Arabidopsis* [51], rice [52], and *Gossypium barbadense* [53]. Terpenoids produced under stress are mostly volatile organic compounds, which can act as pheromones against external organisms or transmit stress signals to the whole plant, thereby inducing other defense responses [48–50]. Conifers have evolved specific defensive traits and strategies that have contributed to their evolutionary diversification and colonization success [54]. The defensive system of Pinaceae, a family that includes pines (*Pinus*) and spruces (*Picea*) among other tree species, relies heavily on resin, a mixture of diterpenes, sesquiterpenes, and monoterpenes that are toxic to herbivores and pathogens [55, 56]. In our study, interestingly, significant GO enrichment was observed in the monoterpenoid biosynthesis pathway (K21374: enriched 27 genes; Fig. 3), indicating that monoterpene compounds synthesis was mainly affected by drought stress. Coniferous resins and essential oils contain a large number of monoterpenes such as alpha-pinene, geraniol, citral, menthol, and iridoids [55, 57, 58]. However, which monoterpene syntheses are affected under drought stress in *P. sylvestris* var. *mongolica* and what their roles are in response to drought stress will be the focus of our further research.

In the correlation analysis, a large number of transcription factors, including *PsNAC1*, were found to be highly correlated with TPS and P450 genes. Several TPS genes (Unigene390_c3_g1 and Unigene390_c1_g1), P450 genes (Unigene4122_c1_g1), and transcription factors (Unigene21862_c0_g1, Unigene9098_c3_g1, Unigene1155_c0_g1, and Unigene2864_c0_g3) were identified as core nodes in the co-expression network (Fig. 4A). These TPS and P450 genes may be regulated by relevant transcription factors, thereby affecting changes in terpenoid synthesis or other physiological responses to drought. In angiosperms, the regulation of TPS and P450 genes by transcription factors has been intensively studied [59]. For example, in kiwifruit, *AaNAC2*, *AaNAC3*, and *AaNAC4* activate the *AaTPS1* promoter, thereby affecting monoterpene compounds synthesis [60]. *CitERF71* activates the terpene synthase gene CitTPS16 involved in the synthesis of E-geraniol in sweet orange fruit [61]. During *A. thaliana* inflorescence, MYC2 activated two sesquiterpene synthase genes (TPS11, TPS21) through the jasmonic acid (JA) and gibberellic acid signaling pathway [62]. In our study, Unigene1155_c0_g1, a MYB transcription factor, had the highest correlation with two TPS genes (Unigene390_c3_g1 and Unigene390_c1_g1) and was a node in the co-expression network. Interestingly, these two TPS genes had completely opposite expression patterns (Fig. 4B) and may not regulate the same terpenes. In addition to their functional diversity, P450s are also involved in stress response under the regulation of transcription factors. In rice, *OsNAC066* contributes positively to rice immunity as a transcriptional activator that activates defense responses by regulating the expression of *OsWRKY62* and a set of cytochrome P450 genes [63]. The rice bHLH transcription factor DPF promotes diterpenoid phytoalexins (DPs) synthesis by binding to cis-acting elements (N-boxes) in CPS2 and CYP99A2 promoter regions, activating CPS2 transcription as well as CYP99A2 genes [64]. Our study showed that key transcription factor, *PsNAC1*, may regulate the expression of a P450 gene Unigene4122_c1_g1 under drought stress as they are significantly negatively correlated and belong to the same co-expression network node. These potential regulatory relationships and specific biological functions require further validation.

## Conclusion

In this study, changes in photosynthetic physiological indices and antioxidant enzyme activities in *P. sylvestris* var. *mongolica* during drought stress and rehydration phases were investigated, and gene expression patterns during these processes were analyzed using transcriptome sequencing. Several key transcription factors (Unigene21862_c0_g1, Unigene9098_c3_g1, Unigene1155_c0_g1, and Unigene2864_c0_g3) were identified through bioinformatics analysis, along with the validation of the overexpressed Unigene9098_c3_g1 (*PsNAC1*) in *Arabidopsis* to enhance drought tolerance. Several TPS genes (Unigene390_c3_g1 and Unigene390_c1_g1) and P450 genes (Unigene4122_c1_g1) were also identified, and the key transcription factors that may regulate these TPS and P450 genes to affect monoterpene synthesis or other biological processes to resist drought stress. These key genes and potential regulatory networks serve as the focus of subsequent drought tolerance studies of *P. sylvestris* var. *mongolica* and provide a theoretical basis for understanding the molecular mechanisms of drought resistance in conifers.

## Methods

### Plant material and drought treatment

Seeds of *P. sylvestris* var. *mongolica* were provided by Qiansongba state-owned forest farm, Fengning County, Hebei Province, China. The seeds were sown to germinate and seedlings were planted in 5 cm pots containing a soil mixture composed of soil and organic matter in a 2:1 v/v ratio. Climatic conditions were controlled in a glasshouse located in Beijing Forestry University in Beijing, China, with a daily average temperature of 28–30 °C (± 2) and relative humidity (RH) of 70–80% during the drought period. After sowing and germination, seedlings were pre-cultured for 2 months prior to drought treatment. Control seedlings were watered daily to field capacity. Physiological parameters were measured for 34 days, with six seedlings subjected to drought treatment and six seedlings maintained under control conditions. Irrigation was withheld for 23 days until the wilting stage was reached, after which seedlings were re-watered to recover hydraulic conductivity. Six control seedlings were continuously irrigated to field capacity throughout the experiment. Leaf samples were collected from each plant for RNA-seq analysis at four time points, with four replicates per time point: 8 days after moderate drought (D1), 23-day drought wilting stage (D2), and 10 days after recovery (D3), with control samples collected at all stages (C1, C2, and C3).

### Photosynthetic parameters measurements

Photosynthetic leaf activities during the drought period, including carbon and water gaseous parameters such as net photosynthesis (AN), stomatal conductance (gs), internal CO_2_ concentration (Ci), and transpiration rate (E), were measured using an open-flow portable photosynthesis system (LI-6400 T, Li-CorInc., Lincoln, NE, USA). The system was equipped with a 6 cm^2^ leaf area chamber with 500 mmol photons m^−2^ s^−1^ of light intensity and with 60% of humidity and humidity, the CO_2_ is stabilized by the CO_2_ supplied to the leaf at 400 ppm or 800 ppm.

### Antioxidants extraction

Antioxidants including peroxidase (POD), and catalase (CAT), polyphenol oxidase (PPO), and malondialdehyde (MDA), were extracted following the manufacturers protocol (Beijing Solarbio Science & Technology, Beijing, China). All four indicators were measured by spectrophotometer method. A 0.1 g of tissue is added to 1 mL of extraction solution for ice bath homogenization and centrifugation for 10 min at 8000 g 4℃, finally, the supernatant was used to determine POD, CAT, PPO and MDA enzyme activity [65–68].

## RNA-seq analysis

Total RNA quantity and purity were assessed using the Nano Photometer spectrophotometer (Implen), and RNA concentration was measured using the Qubit RNA Assay kit in Qubit 2.0 Fluorometer (Life Technologies). RNA integrity was assessed using the RNA Nano 6000 Assay kit of the Bioanalyzer 2100 system (Agilent Technologies). mRNA was fragmented into small pieces using divalent cations under increased temperatures. The cleaved RNA fragments were then reverse-transcribed to create the final cDNA library in accordance with the protocol for the mRNA-Seq sample preparation kit. The average insert size for the paired-end libraries was 200–300 bp. The pooled libraries were sequenced on the BGISEQ (DNBSEQ-T7) platform (2 × 150 bp) using the paired-end module.

### De-novo transcriptome assembly and transcript abundance estimation

Clean data from all samples were *de-novo* assembled using Trinity (version 2.8.5). The initial assembly results were clustered and de-redundant by cd-hit software [69] (version 4.8.1). The longest transcript of each gene was extracted from all transcripts using the get_longest_isoform_seq_per_trinity_gene.pl script provided by Trinity to generate the unigenes. Based on these unigenes, the align_and_estimate_abundance.pl script provided by Trinity was used to invoke a combination of RSEM [70] (version 1.3.3) and Bowtie2 [71] (version 2.3.5) to estimate the transcript abundance of all unigenes in each sample. The transcript abundance data for all samples were used for subsequent differential analysis.

### Functional annotation and differential gene expression analysis

Homologous sequence of all unigenes was performed using BLASTx software (E-values ≤ 1.0 × 10^–5^) and their function annotations were searched against the GO, Nr, COG and KEGG databases. For other unannotated unigenes, we used the TransDecoder program (https://github.com/TransDecoder/TransDecoder) to predict their coding sequence (CDS) and orientation.

The transcript abundance (Transcript per Kilobase per Million mapped reads, TPM) of all unigenes from different samples was analyzed for differential expression using the R package DESeq2 (Version 1.24.0). Differences between treatment and control were evaluated by the form of fold changes, and this study took Log_2_foldchange ≥ 2 (*p* ≤ 0.01) and Log_2_foldchange ≤ -2 (*p* ≤ 0.01) as a criterion for screening upregulated and downregulated DEGs. Gene expression patterns were calculated and standardized using Z-scores transformation [72]. Heatmap of DEGs expression patterns was conducted using the R package pheatmap (version 1.0.12).

### Gene co-expression analysis

All differentially expressed genes (DEGs) were subjected to correlation analysis (Pearson correlation) based on two sets of RNA-Seq data. Genes were selected based on correlation coefficients ≥ 0.8 and *p*-values ≤ 0.01. The CytoNCA tool [73] in Cytoscape was used to analyze differentially expressed hub genes. The top 100 genes were then selected for network analyses, and visualized using Cytoscape 3.8.0 software [74].

### Validation of DEGs

To validate the RNA-Seq data, drought-responsive transcription factors with up- or down-regulation during drought were randomly selected to perform qRT-PCR validation. Seedlings needles from both control and drought were collected and immediately immersed in liquid nitrogen and stored at − 80 °C. The qRT-PCR analysis was performed on the key genes and t-test was used for significance analysis. Primers designed for qRT-PCR and Tubulin is used as housekeeping gene are given in Table S1.

### Vector construction and genetic transformation

The coding regions of *PsNAC1* were inserted into pBI121-GUS plasmids to generate *PsNAC1*-OE overexpression vectors. The overexpression plasmids were introduced into *Agrobacterium tumefaciens* strain GV3101, Agrobacterium culture, injection and tobacco culture methods are as described previously [75]. *Arabidopsis thaliana* ecotype Columbia-0 (Col-0) was transformed by Agrobacterium-mediated floral dip method [76]. Positive transgenic plants were identified by PCR amplification and sequencing, and were maintained to the T2 generation. At least 3 independent lines were selected for further analysis. Primers used for the vector construction are provided in Table S1.

### Supplementary Information

**Supplementary Material 1.**

**Supplementary Material 2.**

**Supplementary Material 3.**

**Supplementary Material 4.**

## Data Availability

The sequenced raw reads generated during the current study have been submitted to the National Center for Biotechnology Information (NCBI) with BioProject ID: PRJNA989562 (https://www.ncbi.nlm.nih.gov/bioproject/?term=PRJNA989562). The expression level (TPM) and functional annotation of all unigenes have been submitted as Table S2 and Table S3. The de novo assembled unigenes in xlsx format have been submitted as Supplementary file 1.
